# Radiotherapy Scheme Effect on PD-L1 Expression for Locally Advanced Rectal Cancer

**DOI:** 10.3390/cells9092071

**Published:** 2020-09-10

**Authors:** Jihane Boustani, Valentin Derangère, Aurélie Bertaut, Olivier Adotevi, Véronique Morgand, Céline Charon-Barra, François Ghiringhelli, Céline Mirjolet

**Affiliations:** 1 Department of Radiation Oncology, Unicancer—Georges-Francois Leclerc Cancer Center, 21079 Dijon, France; jboustani@cgfl.fr (J.B.); vmorgand@cgfl.fr (V.M.); 2 Immunomolecular Therapies in Cancer (TIMC), INSERM UMR1098, 25000 Besancon, France; olivier.adotevi@univ-fcomte.fr; 3 Cancer Biology Research Platform, Unicancer—Georges-Francois Leclerc Cancer Center, 21079 Dijon, France; vderangere@cgfl.fr (V.D.); fghiringhelli@cgfl.fr (F.G.); 4 INSERM UMR 1231, 21079 Dijon, France; 5 Methodology, Data-Management and Biostatistics Unit, Unicancer—Center Georges-Francois Leclerc, 21079 Dijon, France; abertaut@cgfl.fr; 6 Department of Medical Oncology, University Hospital of Besançon, 25000 Besançon, France; 7 Department of Pathology, Unicancer—Georges-Francois Leclerc Cancer Center, 21079 Dijon, France; ccharonbarra@cgfl.fr

**Keywords:** rectal cancer, neoadjuvant radiotherapy, fractionation, PD-L1 expression

## Abstract

In locally advanced rectal cancer, radiotherapy (RT) followed by surgery have improved locoregional control, but distant recurrences remain frequent. Although checkpoint inhibitors have demonstrated objective response in several cancers, the clinical benefit of PD-1/PD-L1 blockade remains uncertain in rectal cancer. We collected data from biopsies and surgical specimens in 74 patients. The main objective was to evaluate the impact of neoadjuvant RT and fractionation on PD-L1 expression. Secondary objectives were to study the relation between PD-L1 expression and tumor regression grade (TRG), progression-free survival (PFS), overall survival (OS), and CD8 TILs infiltration. Median rates of cells expressing PD-L1 pre- and post-RT were 0.15 (range, 0–17) and 0.5 (range, 0–27.5), respectively (*p* = 0.0005). There was no effect of RT fractionation on PD-L1+ cell rates. We found no relation between CD8+ TILs infiltration and PD-L1 expression and no difference between high-PD-L1 or low-PD-L1 expression and TRG. High-to-high PD-L1 expression profile had none significant higher OS and PFS compared to all other groups (*p* = 0.06). Median OS and PFS were higher in biopsies with >0.08 PD-L1+ cells. High-to-high PD-L1 profile and ypT0-2 were significantly associated with higher OS and PFS. This study did not show the differential induction of PD-L1 expression according to fractionation.

## 1. Introduction

The standard treatment of locally advanced rectal cancer (LARC) is neoadjuvant radiotherapy (RT) followed by total mesorectal excision (TME) [1,2]. RT is delivered via either a long-course scheme (i.e., five fractions of 1.8–2 Gy per week for five weeks) or a short-course scheme (i.e., five fractions of 5 Gy in one week (5 × 5 Gy)). Conventionally fractionated long-course RT in combination with chemotherapy followed by surgery after 6–8 weeks has been the predominant treatment [3,4], while short-course RT and surgery within the following week has been commonly used in northern and western Europe. Recently, short-course RT with a delayed surgery has been found to improve tumor regression and to decrease postoperative complications compared to immediate surgery [5]. Preoperative RT can lead to tumor downstaging and to significant reduction of local recurrence, without improving overall survival [3,4,6]. In addition, the complete histological response (pCR) rates vary widely among series between 10% and 30% [7].

In addition to its direct effect on tumor cells, RT is known to elicit an anti-tumor immune response that can lead to tumor eradication [8]. Indeed, RT has been shown to induce a strong lymphocytic infiltration with high densities of CD3+ T cells, CD8+ cytotoxic T cells, and FoxP3+ cells, which are associated with longer disease-free survival and/or overall survival in colorectal cancer [9,10,11,12,13]. On the other hand, RT has been shown to promote immunosuppressive mechanisms. Studies have demonstrated that low doses of fractionated RT can lead to an adaptive upregulation of tumor cell PD-L1 expression that is dependent on CD8+ T-cell production of IFNγ, which may attenuate the efficacy of the anticancer immune response. In preclinical models, fractionated RT with concurrent blockade of PD-1/PD-L1 generated efficient CD8+ T-cell responses that improved local tumor control and long-term survival [14,15]. A difference in PD-L1 expression in tumor tissues was found for various cancers after neoadjuvant therapy [16,17,18]. To date, the expression of PD-L1 in rectal cancer has not been investigated intensively. Despite impressive achievements with immune checkpoint inhibitors, only a small proportion of patients benefit from PD-1/PD-L1 inhibitors. In addition, it is generally acknowledged that PD-L1 expression is one of the most representative predictive biomarkers of PD-1/PD-L1 inhibitors, and strongly PD-L1 positive tumors are expected to respond more robustly to immunotherapy. In non-small cell lung cancer, for instance, PD-1/PD-L1 inhibitors have been recommended as first-line treatments for advanced stage with high PD-L1 expression [19,20]. Thus, PD-L1 levels can affect patients’ outcome. Although radiotherapy is known to induce PD-L1 expression in a multitude of preclinical models, its impact on PD-L1 expression as well as the prognostic and predictive values of the latter in the clinical setting of non-metastatic rectal cancer is less demonstrated and results are less reproducible.

In this study, the main objective was to evaluate the impact of neoadjuvant RT and RT fractionation on PD-L1 expression in patients with LARC. Our secondary objectives were to study the relation between PD-L1 expression and tumor regression grade (TRG), progression-free survival (PFS), and overall survival (OS), as well as the relation between PD-L1 expression and CD8 TILs infiltration.

## 2. Material and Methods

### 2.1. Patients

We retrospectively collected and reviewed the medical records of histologically confirmed LARC patients who underwent a treatment with curative intent. All patients were treated with neoadjuvant short-course RT or long-course concurrent chemoradiotherapy (CRT) followed by TME between 1995 and 2007. Short-course RT was defined as fractions >2 Gy and long-course RT as fractions of ≤2 Gy. Only patients with available pretreatment biopsies and surgical specimens were included. Patients with cancer-tissue specimens fixed using a buffer other than formaldehyde were excluded. The study was conducted in accordance with the Declaration of Helsinki and was approved by the institute review board, the French CCTIRS committee (Comité Consultatif sur le Traitement de l’Information en matière de Recherche et de Santé, Number 13.085), and the CNIL (Commission Nationale de l’Informatique et des Libertés, Number DR.2013.166).

### 2.2. Evaluation of PD-L1 Expression by Immunohistochemistry

**Figure 1:**
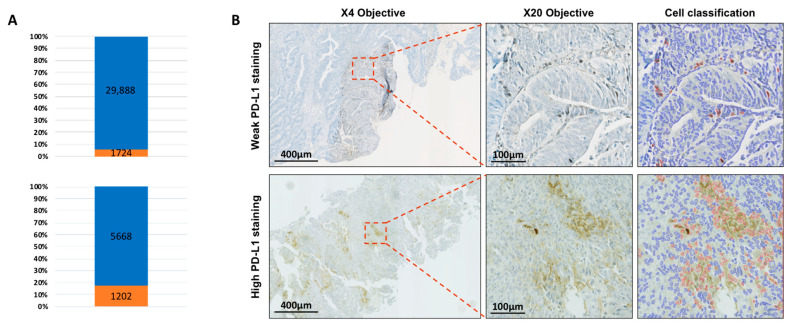
(**A**) Representative histograms of weak (top) and high (bottom) rates of PD-L1 stained cells. Positive cells number are shown in orange, negative cells number in blue. (**B**) Representative weak (top) and high PD-L1 stainings (bottom) at low magnification ((left) scale bar 400 µm) corresponding to patients in (**A**). Higher magnifications are displayed on the center part of the figure (scale bar is 100 µm). Positive cell detection (right) using QuPath (script available in the Supplementary Materials) has been automatically processed. Positive cells are red-outlined while negative cells are blue-outlined.

Diagnostic biopsies and surgical specimens were acquired for each patient. PD-L1 expression was retrospectively detected by immunohistochemistry (IHC) using the monoclonal QR1 anti-PD-L1 antibody (Diagomics), which is used routinely in a clinical situation to evaluate expression of PD-L1 by tumor cells. The slides were independently evaluated by a pathologist (CCB) and a radiation oncology therapist (JB) who were blinded to clinical information. Every slide was digitized using the NDP Nanozoomer scanner HT.2.0 (Hamamatsu Photonics, Shizuoka, Japan). The expression of PD-L1 on cell membranes and cytoplasm was automatically quantified by scripts using the software QuPath [21,22] based on the percentage of stained cells (total tumor and stromal cells) in three regions per slide. The results were expressed as the rates of cells expressing PD-L1 (Figure 1).

### 2.3. TILs Assessment

CD8+ TILs were retrospectively analyzed in collected formalin-fixed paraffin of surgical samples and biopsies. Immunohistochemistry used monoclonal antibodies against T-cell marker CD8 (dilution 1/200, clone C8/144B, Dako, France) using the same portal as previously described. Every slide was digitized using the NDP Nanozoomer scanner (Hamamatsu Photonics, Shizuoka, Japan). CD8+ cells were quantified using 20× magnification on at least three distinct and representative fields of 0.46 mm^2^ at the level of tumor, tumor regression, or tumor site (in cases with a complete response) for surgical samples. Histopathologic evaluation of TILs was performed by a pathologist and a biologist who were blinded to clinical information as previously described [13]. Using the generalized kappa test, the biologist evaluation was validated by a substantial agreement [13]. Mean values of these three fields were analyzed. Using criteria described by Denkert et al. [23], intraepithelial TILs (iTILs) were defined as the number of lymphocytes per field in direct contact with tumor cells, whereas stromal TILs (sTILs) were defined as the number of lymphocytes per field in the tumor stroma.

### 2.4. Assessment of Response to Neoadjuvant Radiotherapy

Surgical specimens were assessed for tumor response to neoadjuvant treatment. Tumor response rates were analyzed according to the Mandard Tumor Regression Grade (TRG) as follows [24]: 1, no residual cancer cells; 2, rare residual cancer cells; 3, predominant fibrosis with increased number of residual cancer cells; 4, residual cancer outgrowing fibrosis; and 5, no regressive change

### 2.5. Statistical Analyses

The main objective was to evaluate the impact of neoadjuvant RT and RT fractionation on the rate of cells expressing PD-L1 in patients with LARC. Our secondary objectives were to study the relation between PD-L1+ cells rate and TRG, PFS and OS, as well as the relation between PD-L1+ cells rate and CD8 TILs infiltration. Quantitative variables were described using mean with standard deviation (SD) or median with range and compared using Student test or Wilcoxon test in case of normal distribution or not, respectively. Qualitative variables were described as frequencies and percentages and compared between groups using Chi-square test or Fisher exact test. The rate of PD-L1+ cells pre-RT and post-RT were compared using the Wilcoxon signed rank test. Median follow-up was calculated according to the reverse Kaplan–Meier method. OS and PFS survival curves and median survival times (with their 95% confidence interval) were estimated using the Kaplan–Meier method. The log-rank test was used to compare survival curves. Univariate and multivariate Cox proportional hazards regressions were used to estimate hazard ratio (HR) with its 95% CI. Variables with a *p* < 0.20 in univariate analysis were included in the multivariate model. Correlations between all variables included in the model were explored. Tests were two-sided and a *p*-value less than 5% was considered statistically significant. All analyses were performed using SAS software version 9.4.

## 3. Results

### 3.1. Patients, Tumor, and Treatment Characteristics

Patients’ clinicopathologic characteristics and treatment modalities are summarized in Table 1. There were 74 patients included with a median age of 69 years (range, 29–85) and a majority of men (63.5%). Most patients had a moderately (51%) to highly (43%) differentiated adenocarcinoma of the lower (35%) or middle (46%) rectum. There were 58 (88%) cT3 tumors, and 27 (44%) patients had cN+ disease. On surgical specimens, three patients (4%) had ypT1 disease, 16 patients (22%) were classified ypT2, 46 patients (62%) were ypT3, five patients (7%) were ypT4, and 23 patients (31%) had pathologically positive nodes. Four patients (5%) had no residual tumor (ypT0), and 49 patients (68%) were classified ypN0. Pathologic tumor regression of grade 1–3 was reported in 46 patients (63%). Short-course RT was performed in 27 patients (36.5%), and long-course RT in 47 patients (63.5%). Only 19 patients (27%) had concurrent chemotherapy which combined 5-fluorouracil and folic acid in most of the cases (63%). Adjuvant chemotherapy was administered in 17 patients (24%). No neoadjuvant chemotherapy was given.

### 3.2. Effect of Short-Course versus Long-Course RT on PD-L1+ Cells

The median rates of cells expressing PD-L1 pre-RT and post-RT were 0.15 (range, 0.0–17.0) and 0.5 (range, 0.0–27.5), respectively (*p* = 0.0005). The median values were used as cut offs stratifying the high and low levels of PD-L1+ cells. Table 1 represents the association between clinicopathologic characteristics and PD-L1+ cells at baseline. The rate of PD-L1+ cells at baseline differed significantly according to the tumor location. For patients with low level of PD-L1+ cells, 19% of tumors were located within 5 cm from the anal verge, 61% between 5 and 10 cm, and 19% >10 cm from the anal verge vs. 50%, 31%, and 19%, respectively, for patients with high level PD-L1+ cells (*p* = 0.01). This was not found after treatment on surgical specimens (Table 1).

The effect of short-course versus long-course RT on PD-L1+ cells is shown in Table 2. The median PD-L1+ cells rate on biopsy was 0.1 (range, 0.0–17.0) in patients receiving long-course RT and 0.2 (range, 0.0–4.8) in patients receiving short-course RT (*p* = 0.56). The median PD-L1+ cells rate on surgical specimen was 0.5 (range, 0.0–27.5) in patients receiving long-course RT and 0.6 (range, 0.0–5.9) in patients receiving short-course RT (*p* = 0.47). The median change in the level of PD-L1+ cells rate was 0.2 (range, −17.0–26.0) after long-course RT and 0.1 (range, −4.1–3.1) after short-course RT (*p* = 0.53).

### 3.3. Relation between CD8 TILs Infiltration and PD-L1+ Cells

We evaluated the relation between CD8 TILs and PD-L1+ cells (Table 3).

At baseline, CD8 TILs data were available for 53 patients. The median CD8 TILs was 44.5 (range, 1.3–113.0) in PD-L1 ≤ 0.15 and 42.8 (range, 0.3–290.7) in PD-L1 > 0.15 (*p* = 0.65). Intraepithelial CD8 TILs and stromal CD8 TILs did not differ significantly between low PD-L1 and high PD-L1 biopsies.

After treatment, CD8 TILs data were available for 73 patients. The median CD8 TILs was 55.8 (range, 1.7–273.7) in PD-L1 ≤ 0.5 and 51.5 (range, 4.0–392.8) in PD-L1 >0.5 (*p* = 0.99).

Intraepithelial CD8 TILs and stromal CD8 TILs did not differ significantly between low PD-L1 and high PD-L1 surgical specimens.

### 3.4. Survival and Tumor Regression Grade Analysis

**Figure 2:**
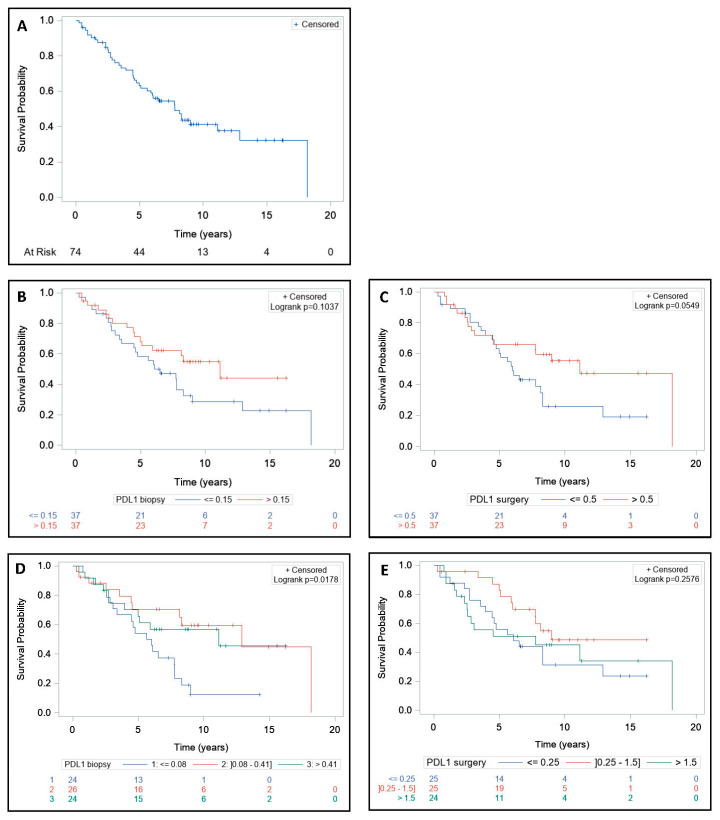
Impact of PD-L1 expression on overall survival before and after treatment. Patients (*n* = 74) were treated with neoadjuvant short-course radiotherapy or long-course concurrent chemoradiotherapy followed by surgery. Biopsies and surgical specimens were collected and PD-L1 expression was retrospectively detected by immunohistochemistry using the monoclonal QR1 anti-PD-L1 antibody (Diagomics). The expression of PD-L1 on cell membranes and cytoplasms was automatically quantified by scripts using the software QuPath based on the percentage of stained cells (total tumor and stromal cells) in three regions per slide. The overall survival (OS) in the entire cohort (**A**); OS according to median PD-L1+ cells rate on biopsies (**B**); OS according to median PD-L1+ cells rate on surgical specimens (**C**); OS according to first, second, and third terciles on biopsies (**D**); and OS according to first, second, and third terciles on surgical specimens (**E**). OS curves were estimated using the Kaplan–Meier method. The log-rank test was used to compare survival curves.

**Figure 3:**
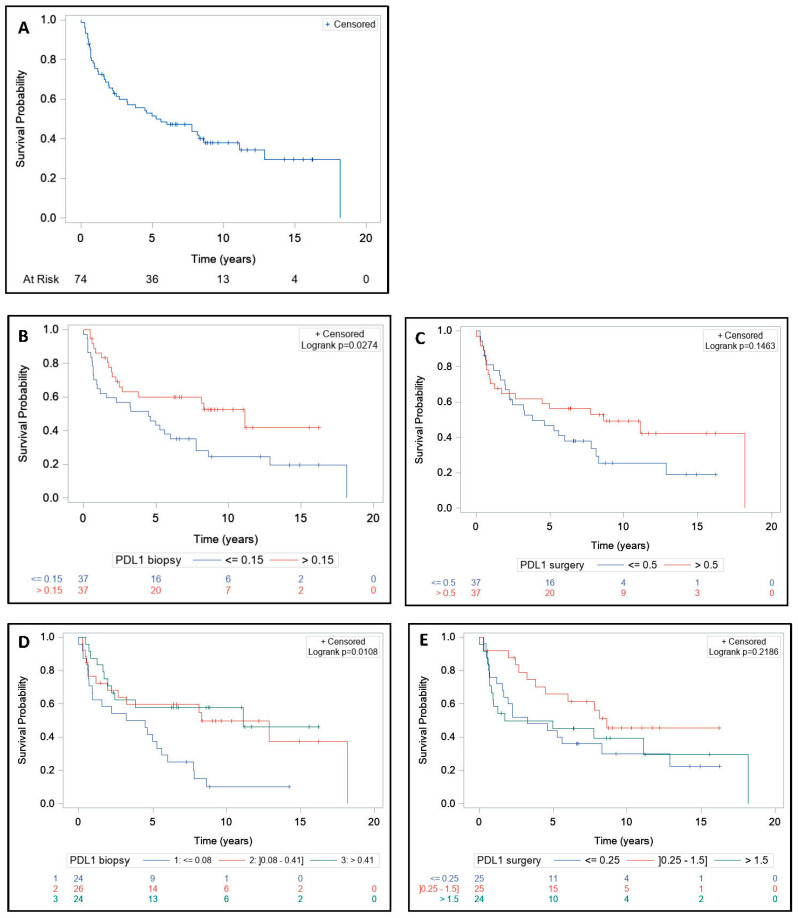
Impact of PD-L1 expression on progression-free survival before and after treatment. Patients (*n* = 74) were treated with neoadjuvant short-course radiotherapy or long-course concurrent chemoradiotherapy followed by surgery. Biopsies and surgical specimens were collected and PD-L1 expression was retrospectively detected by immunohistochemistry using the monoclonal QR1 anti-PD-L1 antibody (Diagomics). The expression of PD-L1 on cell membranes and cytoplasms was automatically quantified by scripts using the software QuPath based on the percentage of stained cells (total tumor and stromal cells) in three regions per slide. The progression-free survival (PFS) in the entire cohort (**A**); PFS according to median PD-L1+ cells rate on biopsies (**B**); PFS according to median PD-L1+ cells rate on surgical specimens (**C**); PFS according to first, second, and third terciles on biopsies (**D**); and PFS according to first, second, and third terciles on surgical specimens (**E**). PFS curves were estimated using the Kaplan–Meier method. The log-rank test was used to compare survival curves.

**Figure 4:**
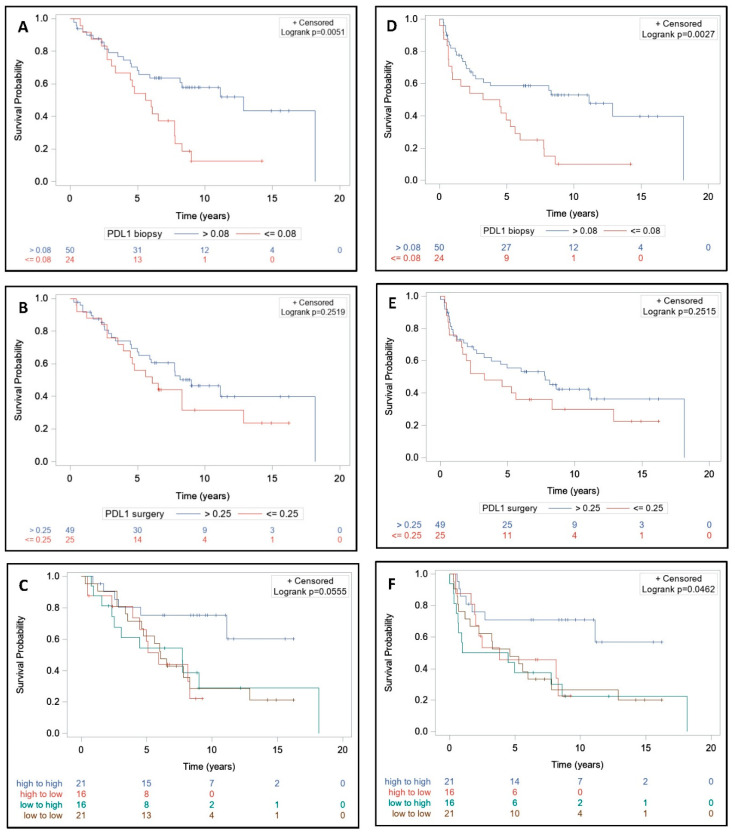
Impact of PD-L1 expression on overall survival (OS) and progression-free survival (PFS) before and after treatment. Patients (*n* = 74) were treated with neoadjuvant short-course radiotherapy or long-course concurrent chemoradiotherapy followed by surgery. Biopsies and surgical specimens were collected and PD-L1 expression was retrospectively detected by immunohistochemistry using the monoclonal QR1 anti-PD-L1 antibody (Diagomics). The expression of PD-L1 on cell membranes and cytoplasms was automatically quantified by scripts using the software QuPath based on the percentage of stained cells (total tumor and stromal cells) in three regions per slide. The OS and PFS according to the first tercile of PD-L1+ cells rate on biopsies (**A**,**D**); OS and PFS according to the first tercile of PD-L1+ cells rate on surgical specimens (**B**,**E**); and OS and PFS according to the first tercile of PD-L1+ cells rate on PDL1 expression evolution before and after treatment (**C**,**F**), respectively. OS and PFS curves were estimated using the Kaplan–Meier method. The log-rank test was used to compare survival curves.

Median follow-up was nine years (range, 0.3–18.2), median OS was 7.8 years (range, 5.1–12.9) (Figure 2A), and median PFS was 5.6 years (range, 2.5–8.6) (Figure 3A). Median OS was 11.1 years (range, 5.0-NE) in >0.15 PD-L1+ cells group and 6.5 years (range, 3.6–8.3) in ≤0.15 PD-L1+ cells group (*p* = 0.10) (Figure 2B). Median OS was significantly lower in the first tercile, which corresponds to biopsies with ≤0.08 PD-L1+ cells (*p* = 0.01) (Figure 2D and Figure 4A). Median OS was 11.1 years (range, 4.5-NE) in >0.5 PD-L1+ cells group and 6 years (range, 4.6–8.3) in ≤0.5 PD-L1+ cells group on surgical specimens (*p* = 0.05) (Figure 2C). Median OS did not differ significantly among the first, second, and third tercile groups (*p* = 0.25) (Figure 2E and Figure 4B).

Median PFS was significantly higher in >0.15 PD-L1+ cells group at baseline (*p* = 0.02) (Figure 3B). Median PFS was significantly lower in the first tercile, which corresponds to biopsies with ≤0.08 PD-L1+ cells (*p* = 0.01) (Figure 3D and Figure 4D). Median PFS was 8.6 years (range, 1.7-NE) in >0.5 PD-L1+ cells group and 3.8 years (range, 2.0–7.8) in ≤0.5 PD-L1+ cells group on surgical specimens (*p* = 0.14) (Figure 3C). Median PFS did not differ significantly among the first, second, and third tercile groups (*p* = 0.21) (Figure 3E and Figure 4E).

Subgroups with low-to-low, low-to-high, high-to-low, and high-to-high expression before and after RT were specified. The high-to-high PD-L1 expression profile had a higher OS compared to all other groups but the difference was not statistically significant (*p* = 0.06) (Figure 4C). High-to-high PD-L1 profile and ypT0-2 were significantly associated with a longer OS in both univariate and multivariate analysis (Table 4). The high-to-high PD-L1 expression profile had a statistically significant higher PFS compared to all other groups (*p* = 0.04) (Figure 4D). High-to-high PD-L1 profile and ypT0-2 were significantly correlated to PFS in both univariate and multivariate analysis (Table 5).

In Table S1, TRG is shown according to PD-L1 expression at baseline and after treatment. There was no statistically significant difference between high-PD-L1 or low-PD-L1 expression and TRG at baseline or after treatment. There was no difference between subgroups of PD-L1 expression profile.

## 4. Discussion

Studies have shown that RT can modify the tumor microenvironment by inducing both immunostimulation and immunosuppression. The upregulation of PD-L1 expression in cancer cells interferes with the effector functions of T cells. Since the PD-1/PD-L1 interaction represents one of the major mechanisms of cancer immune escape, which leads to treatment failure, the evaluation of PD-L1 expression in response to RT is important for the establishment of the optimal combination strategy. In the present study, the mean rate of PD-L1+ cells was 1.1%, similar to rates reported in other studies. Hecht et al. reported that the percentage of tPD-L1 high expression was 2.1% in rectal cancer before CRT [25]. Lee et al. reported that high tPD-L1 expression was identified in 4.8% of the total cohort, consisting of 2.2% in the mismatch-repair proficient group. In our analysis, we found that the proportion of PD-L1+ cells increased after neoadjuvant RT, which is line with other studies [16,25]. Interestingly, the increase seemed long-lasting since the median interval between RT and surgery was nearly six weeks. Surprisingly, tumors located in the lower rectum expressed a higher rate of PD-L1+ cells, whereas tumors of the middle rectum expressed a lower rate of PD-L1+ cells. On the contrary, Lim et al. found that tumor location ≥6 cm from the anal verge was associated with higher pre-CRT PD-L1 expression [26]. Tumor location ≤ 6 cm was an independent negative prognostic factor for OS^22^. Berntsson et al. evaluated the prognostic impact of PD-L1 expression in relation to primary tumor location [27]. They found that high PD-L1 expression on tumor-infiltrating immune cells, but not tumor-specific PD-L1 expression, was associated with a prolonged OS in tumor of the right colon and the left colon but not in rectal cancer [27]. However, the PD-L1 expression was not evaluated according to the tumor location in the rectum in their study. 

In our cohort, survival rates were consistent with previously published outcomes in LARC patients treated with neoadjuvant RT [3,4,28]. When the study population was classified according to PD-L1 expression profile, a high-to-high PD-L1 profile was associated with better survival outcomes than the other groups. Multivariate model showed a significant association between high baseline PD-L1 expression and high OS and PFS, with the highest outcomes observed in the high-to-high PD-L1 subgroup. This paradoxical observation, in which the expression of an immunosuppressive checkpoint correlates with improved outcomes, has been previously described. In 112 pair-matched LARC patients treated with CRT, a high tumor PD-L1 before and after CRT was associated with improved disease-free survival and OS [29]. A strong PD-L1 expression in mismatch repair-proficient colorectal cancer was significantly associated with less aggressive tumors and significantly improved survival [28]. Hecht et al. reported that combined low PD-L1 expression on tumor and inflammatory cells was an independent negative prognostic marker for OS and high PD-L1 expression in biopsies tended to be associated with improved disease-free survival and OS [25]. One plausible explanation is that PD-L1 expression might reflect the presence of an endogenous antitumor immunity and the balance of the host’s immune response and negative feedback will determine the outcome [30]. It has been previously shown that the type, density, and location of immune cells within the tumor site could predict survival in colorectal cancer more accurately than the classical TNM system [31]. This finding led to classifying tumors between hot and cold. Typically, hot tumors are highly infiltrated with T cells and respond favorably to anti-tumor therapy [32]. A high expression of PD-L1 on tumor-associated immune cells has also been described as a characteristic of hot tumors [33]. Thus, in our study, a high PD-L1 expression might be the reflection of a hot tumor, which might further explain better long-term survival. Conversely, Lim et al. reported worse survival outcomes in high-to-high PD-L1 profile [26]. These contradictory results might be due to inherent confounding factors, such as the use of a variety of different antibodies for immunohistochemistry, inconsistent cut-off values, as well as tumor and patient heterogeneity. 

We found no correlation between CD8 TILs infiltration and PD-L1 expression neither at baseline nor after surgery. However, one limitation was the small number of CD8 TIL density assessment available in only 53 patients at baseline and the relatively small size of the entire cohort. Lim et al. performed a paired analysis of pre- and post-CRT rectal cancer and demonstrated that both PD-L1 expression and the density of CD8 TILs markedly increased after preoperative CRT [16]. Patients with a consistently high level of PD-L1 expression before and after CRT experienced less of an increase in CD8+ TILs than those in other groups, and low-to-low density of CD8 TILs was associated with worse survival outcomes [16]. On the other hand, Ogura et al. found that, before CRT, stromal CD8 cell density was significantly higher in patients with high PD-L1 expression on stromal immune cells compared with low PD-L1 expression but stromal CD8 cell density was not significantly different [32]. After CRT, high PD-L1 expression on stromal immune cells was associated with high stromal CD8 cell density. High CD8 cell density in tumor areas before CRT was associated with better disease-free survival in patients with high PD-L1 expression and not in patients with low PD-L1 expression on stromal immune cells [34].

We found no effect of short-course versus long-course RT on PD-L1+ cells. Shao et al. reported, in a retrospective study of 68 rectal cancer patients treated with neoadjuvant RT, that tumor cells PD-L1+ was significantly correlated to short-course RT, suggesting that PD-L1 expression might be regulated by modifying the RT scheme [35]. This would allow the addition of an immune checkpoint inhibitor targeting PD-1/PD-L1 axis in the adjuvant setting. However, more clinical studies are required to demonstrate the same findings.

We found no correlation between TRG and PD-L1+ cells. Only one study found that PD-L1 expression on stromal immune cells significantly increased after CRT only in patients with no or moderate regression (with dominant tumor mass with obvious fibrosis or dominantly fibrotic changes with few tumor cells) [34].

Our study has several limitations. First, this was a retrospective study. Second, the relatively small number of patients included in the analysis with heterogeneous treatments. Third, we used only one antibody to evaluate PD-L1 staining and we did not report the intensity of staining. Fourth, the PD-L1 expression on stromal immune cells and tumor cells was not evaluated separately. Moreover, the comparison of small biopsy specimens and larger surgical resection specimens was challenging since the expression of PD-L1 is known to be heterogeneous throughout the tumor. Finally, we did not study the PD-L1 expression during CRT which could be interesting especially when considering the ideal timing for concurrent association between RT and immune checkpoint inhibitor.

## 5. Conclusions

In patients with locally advanced rectal cancer treated with neoadjuvant radiotherapy, we demonstrated an increase in PD-L1 expression after radiotherapy, but we did not find a differential induction of PD-L1 expression according to fractionation. Patients with high-to-high PD-L1 expression had better outcomes, suggesting that patients with “hot tumors” are more likely to respond to treatment. Future work needs to evaluate whether adding anti-PD-1/PD-L1 antibodies to neoadjuvant radiotherapy in this setting can enhance anti-tumor immune responses and translate into clinical benefit. In addition, it will be interesting to understand the impact of combining radiotherapy with anti-PD-1/PD-L1 antibodies on the immune response to convert an immune cold into a hot tumor. 

## Figures and Tables

**Table 1:** **Patients, tumor, and treatment characteristics for the entire cohort (Column Cohort).** Characteristics were compared according to the median PD-L1+ cells rate on biopsy (column pre-treatment) and surgical specimen (column post-treatment).

	Cohort	Pre-Treatment			Post-Treatment		
		PDL1 ≤ 0.15	PDL1 > 0.15	*p* Value	PDL1 ≤ 0.5	PDL1 > 0.5	*p* Value ^4^
**Age (Years)**				0.14			0.39
Mean (SD ^1^)	68.1 (10.0)	66.2 (10.7)	69.9 (9.0)		67.0 (11.0)	69.1 (9.0)	
Median [range]	69.0 [29.0–85.0]	68.0 [29.0–81.0]	70.0 [48.0–85.0]		69.0 [29.0–84.0]	70.0 [43.0–85.0]	
**Gender**				0.09			0.22
Male	47 (63.5%)	27 (73.0%)	20 (54.1%)		26 (70.3%)	21 (56.8%)	
Female	27 (36.5%)	10 (27.0%)	17 (45.9%)		11 (29.7%)	16 (43.2%)	
**Tumor Distance from Anal Verge (mm)**				0.08			0.15
N	72	36	36		37	35	
Mean (SD)	6.2 (3.8)	6.9 (3.8)	5.4 (3.8)		6.5 (3.5)	5.7 (4.1)	
Median [range]	6.0 [0.0–18.0]	6.5 [0.0–18.0]	4.5 [0.0–15.0]		7.0 [0.0–15.0]	6.0 [0.0–18.0]	
**Tumor Distance from Anal Verge (mm)**				**0.01**			0.36
<5 cm	25 (34.7%)	7 (19.4%)	18 (50.0%)		10 (27.0%)	15 (42.9%)	
[5–10] cm	33 (45.8%)	22 (61.1%)	11 (30.6%)		19 (51.4%)	14 (40.0%)	
≥10 cm	14 (19.4%)	7 (19.4%)	7 (19.4%)		8 (21.6%)	6 (17.1%)	
Missing	2	1	1		0	2	
**Histopathological Subtype**				0.21			0.74
Poorly differentiated adenocarcinoma	2 (2.9%)	2 (5.6%)	0 (0.0%)		1 (2.9%)	1 (2.8%)	
Moderately differentiated adenocarcinoma	36 (51.4%)	21 (58.3%)	15 (44.1%)		20 (58.8%)	16 (44.4%)	
Highly differentiated adenocarcinoma	30 (42.9%)	12 (33.3%)	18 (52.9%)		12 (35.3%)	18 (50.0%)	
Other	2 (2.9%)	1 (2.8%)	1 (2.9%)		1 (2.9%)	1 (2.8%)	
Missing	4	1	3		3	1	
**cT Stage**				0.59			0.70
T2	2 (3.0%)	0 (0.0%)	2 (6.1%)		1 (3.2%)	1 (2.9%)	
T3	58 (87.9%)	30 (90.9%)	28 (84.8%)		26 (83.9%)	32 (91.4%)	
T4	6 (9.1%)	3 (9.1%)	3 (9.1%)		4 (12.9%)	2 (5.7%)	
Missing	8	4	4		6	2	
**cN Stage**				0.88			0.37
N0	34 (55.7%)	17 (56.7%)	17 (54.8%)		19 (61.3%)	15 (50.0%)	
N+	27 (44.3%)	13 (43.3%)	14 (45.2%)		12 (38.7%)	15 (50.0%)	
Missing	13	7	6		6	7	
**cM Stage**				1.00			1.00
M0	66 (95.7%)	33 (97.1%)	33 (94.3%)		34 (94.4%)	32 (97.0%)	
M+	3 (4.3%)	1 (2.9%)	2 (5.7%)		2 (5.6%)	1 (3.0%)	
Missing	5	3	2		1	4	
**Radiation Dose Per Fraction (Gy)**				0.46			0.22
≤2	47 (63.5%)	25 (67.6%)	22 (59.5%)		26 (70.3%)	21 (56.8%)	
>2	27 (36.5%)	12 (32.4%)	15 (40.5%)		11 (29.7%)	16 (43.2%)	
**Chemotherapy**				0.11			0.88
No	44 (60.3%)	19 (51.4%)	25 (69.4%)		22 (59.5%)	22 (61.1%)	
Yes	29 (39.7%)	18 (48.6%)	11 (30.6%)		15 (40.5%)	14 (38.9%)	
Missing	1	0	1		0	1	
**Neoadjuvant Chemotherapy**							
No	68 (100.0%)	34 (100.0%)	34 (100.0%)		33 (100.0%)	35 (100.0%)	
Missing	6	3	3		4	2	
**Concurrent Chemotherapy**				0.73			0.46
No	52 (73.2%)	25 (71.4%)	27 (75.0%)		25 (69.4%)	27 (77.1%)	
Yes	19 (26.8%)	10 (28.6%)	9 (25.0%)		11 (30.6%)	8 (22.9%)	
Missing	3	2	1		1	2	
**Type of Concurrent Chemotherapy**				0.82			0.92
5FU ^2^	1 (5.3%)	0 (0.0%)	1 (11.1%)		1 (9.1%)	0 (0.0%)	
Xeloda	3 (15.8%)	2 (20.0%)	1 (11.1%)		2 (18.2%)	1 (12.5%)	
Folinic acid + 5FU	12 (63.2%)	7 (70.0%)	5 (55.6%)		7 (63.6%)	5 (62.5%)	
Folfox	1 (5.3%)	0 (0.0%)	1 (11.1%)		0 (0.0%)	1 (12.5%)	
Cisplatine + 5FU	2 (10.5%)	1 (10.0%)	1 (11.1%)		1 (9.1%)	1 (12.5%)	
**Adjuvant Chemotherapy**				0.20			0.78
No	55 (76.4%)	26 (70.3%)	29 (82.9%)		27 (75.0%)	28 (77.8%)	
Yes	17 (23.6%)	11 (29.7%)	6 (17.1%)		9 (25.0%)	8 (22.2%)	
Missing	2	0	2		1	1	
**Type of Adjuvant Chemotherapy**				1.00			0.56
Folinic acid + 5 FU	9 (52.9%)	6 (54.5%)	3 (50.0%)		5 (55.6%)	4 (50.0%)	
Folfox	4 (23.5%)	2 (18.2%)	2 (33.3%)		1 (11.1%)	3 (37.5%)	
Other	4 (23.5%)	3 (27.3%)	1 (16.7%)		3 (33.3%)	1 (12.5%)	
**Time between End of Radiotherapy and Surgery (Days)**				0.92			0.11
N	74	37	37		37	37	
Mean (SD)	43.4 (35.3)	46.6 (48.8)	40.2 (11.0)		42.9 (13.2)	43.9 (48.5)	
Median [Range]	40.0 [4.0–319.0]	42.0 [4.0–319.0]	40.0 [19.0–65.0]		42.0 [15.0–77.0]	39.0 [4.0–319.0]	
**Conservative Surgery**				0.07			0.18
No	32 (43.8%)	12 (33.3%)	20 (54.1%)		13 (36.1%)	19 (51.4%)	
Yes	41 (56.2%)	24 (66.7%)	17 (45.9%)		23 (63.9%)	18 (48.6%)	
Missing	1	1	0		1	0	
**Margins**				1.00			0.35
R0	69 (94.5%)	34 (94.4%)	35 (94.6%)		36 (97.3%)	33 (91.7%)	
R1	4 (5.5%)	2 (5.6%)	2 (5.4%)		1 (2.7%)	3 (8.3%)	
Missing	1	1	0		0	1	
**pN+**				0.11			0.67
No	49 (67.1%)	21 (58.3%)	28 (75.7%)		24 (64.9%)	25 (69.4%)	
Yes	24 (32.9%)	15 (41.7%)	9 (24.3%)		13 (35.1%)	11 (30.6%)	
Missing	1	1	0		0	1	
**ypT Stage**				0.69			0.40
0	4 (5.4%)	2 (5.4%)	2 (5.4%)		3 (8.1%)	1 (2.7%)	
1	3 (4.1%)	1 (2.7%)	2 (5.4%)		1 (2.7%)	2 (5.4%)	
2	16 (21.6%)	7 (18.9%)	9 (24.3%)		5 (13.5%)	11 (29.7%)	
3	46 (62.2%)	23 (62.2%)	23 (62.2%)		25 (67.6%)	21 (56.8%)	
4	5 (6.8%)	4 (10.8%)	1 (2.7%)		3 (8.1%)	2 (5.4%)	
**ypN Stage**				0.29			0.43
0	49 (68.1%)	22 (61.1%)	27 (75.0%)		23 (63.9%)	26 (72.2%)	
1	15 (20.8%)	8 (22.2%)	7 (19.4%)		7 (19.4%)	8 (22.2%)	
2	8 (11.1%)	6 (16.7%)	2 (5.6%)		6 (16.7%)	2 (5.6%)	
Missing	2	1	1		1	1	
**TRG ^3^**				0.58			0.21
1	4 (5.5%)	2 (5.6%)	2 (5.4%)		3 (8.1%)	1 (2.8%)	
2	18 (24.7%)	9 (25.0%)	9 (24.3%)		11 (29.7%)	7 (19.4%)	
3	24 (32.9%)	9 (25.0%)	15 (40.5%)		11 (29.7%)	13 (36.1%)	
4	23 (31.5%)	13 (36.1%)	10 (27.0%)		12 (32.4%)	11 (30.6%)	
5	4 (5.5%)	3 (8.3%)	1 (2.7%)		0 (0.0%)	4 (11.1%)	
Missing	1	1	0		0	1	

^1^ Standard deviation; ^2^ 5 fluorouracil; ^3^ tumor regression grade. ^4^ Quantitative variables were compared using Student test or Wilcoxon test in case of normal distribution or not, respectively. Qualitative variables were compared between groups using Chi-square test or Fisher exact test. Tests were two-sided and a *p*-value less than 5% was considered statistically significant.

**Table 2:** Comparison of PD-L1+ cells rate according to short-course (>2 Gy) and long-course radiotherapy (≤2 Gy).

	≤2 Gy	>2 Gy	*p* Value ^4^
**PD-L1+ Cells Rate on Biopsy**			0.56
*n* ^1^	47	27	
Mean (SD ^2^)	1.1 (2.9)	1.1 (1.5)	
Median [range]	0.1 [0.0–17.0]	0.2 [0.0–4.8]	
**Median PD-L1+ Cells Rate on Biopsy**			0.46
≤0.15	25 (53.2%)	12 (44.4%)	
>0.15	22 (46.8%)	15 (55.6%)	
**PD-L1+ Cells Rate on Surgical Specimen**			0.33
*n*	47	27	
Mean (SD)	1.8 (4.3)	1.4 (1.6)	
Median [range]	0.5 [0.0–27.5]	0.6 [0.0–5.9]	
**Median PD-L1+ Cells Rate on Surgical Specimen**			0.22
≤0.5	26 (55.3%)	11 (40.7%)	
>0.5	21 (44.7%)	16 (59.3%)	
**Delta PD-L1 ^3^**			0.52
N	47	27	
Mean (SD)	0.7 (4.9)	0.4 (1.8)	
Median [range]	0.2 [-17.0–25.9]	0.1 [-4.1–3.1]	

^1^ number of patients; ^2^ standard deviation; ^3^ Delta PD-L1 represents PD-L1+ cells rate after treatment minus PD-L1+ cells rate before treatment; ^4^ Quantitative variables were compared using Student test or Wilcoxon test in case of normal distribution or not, respectively. Qualitative variables were compared between groups using Chi-square test or Fisher exact test. Tests were two-sided and a *p*-value less than 5% was considered statistically significant.

**Table 3:** Relation between CD8 TILs infiltration and PD-L1+ cells median values at pre-treatment (biopsy) and post-treatment (surgical specimen).

	**PD-L1 Biopsy ≤ 0.15** **(*n* = 27)**	**PD-L1 Biopsy > 0.15** **(*n* = 26)**	***p* Value ^3^**
**CD8 TILs ^1^ on Biopsy**			0.65
Mean (SD ^2^)	47.4 (32.3)	68.8 (70.4)	
Median [range]	44.5 [1.3–113.0]	42.8 [0.3–290.7]	
**Intraepithelial CD8 TILs on Biopsy**			0.62
Mean (SD)	10.5 (13.4)	14.8 (19.4)	
Median [range]	6.0 [0.0–50.9]	6.0 [0.0–87.2]	
**Stromal CD8 TILs on Biopsy**			0.69
Mean (SD)	36.9 (23.9)	54.1 (54.3)	
Median [range]	37.8 [1.3–87.3]	34.2 [0.3–203.5]	
	**PD-L1 Surgery ≤ 0.5** **(*n* = 37)**	**PD-L1 Surgery > 0.5** **(*n* = 36)**	***p* Value**
**CD8 TILs on Surgical Specimen**			0.99
Mean (SD)	63.6 (49.2)	71.0 (71.6)	
Median [range]	55.8 [1.7–273.7]	51.5 [4.0–392.8]	
**Intraepithelial CD8 TILs on Surgical Specimen**			0.17
Mean (SD)	6.6 (10.9)	11.2 (15.6)	
Median [range]	1.6 [0.0–49.2]	4.4 [0.0–65.9]	
**Intraepithelial CD8 TILs on Surgical Specimen**			0.82
Mean (SD)	57.0 (44.5)	59.8 (60.9)	
Median [range]	49.2 [1.7–251.8]	46.9 [2.8–353.6]	

^1^ tumor infiltrating lymphocytes; ^2^ standard deviation; ^3^ Quantitative variables were compared using Student test or Wilcoxon test in case of normal distribution or not, respectively. Qualitative variables were compared between groups using Chi-square test or Fisher exact test. Tests were two-sided and a *p*-value less than 5% was considered statistically significant.

**Table 4:** Relation between clinical variables at baseline and after treatment and overall survival.

		Univariate Analysis			Multivariate Analysis		
		HR ^3^	95% CI ^4^	*p* Value ^5^	HR	95% CI	*p* Value ^5^
**Gender**	***n* = 74**			0.93			
Female vs Male		1.025	[0.542–1.940]				
**Age (Years)**	***n* = 74**			0.97			
>69 vs ≤69		1.012	[0.543–1.884]				
**Tumor Distance from Anal Verge (cm)**	***n* = 72**			0.37			
≥10 cm vs <5 cm		1.398	[0.570–3.427]				
[5,6,7,8,9,10] cm vs <5 cm		1.667	[0.811–3.425]				
**Histological Subtype**	***n* = 70**			0.79			
Highly differentiated vs Poorly or Moderately differentiated adenocarcinoma		0.827	[0.428–1.597]				
Other vs Poorly or Moderately differentiated adenocarcinoma		1.211	[0.284–5.170]				
**cN Stage**	***n* = 61**			0.39			
N+ vs N0		1.346	[0.678–2.674]				
**Radiation Dose Per Fraction (Gy)**	***n* = 74**			0.10			
>2 vs ≤2		1.686	[0.899–3.163]				
**Chemotherapy**	***n* = 73**			0.82			
Yes vs No		1.074	[0.577–1.996]				
**Concurrent Chemotherapy**	***n* = 71**			0.88			
Yes vs No		0.949	[0.470–1.915]				
**Adjuvant Chemotherapy**	***n* = 72**			0.24			
Yes vs No		1.499	[0.763–2.945]				
**Time between End of Radiotherapy and Surgery (Days)**	***n* = 74**			0.24			
>40 vs ≤40		1.437	[0.775–2.663]				
**Conservative Surgery**	***n* = 73**			0.61			
Yes vs No		1.175	[0.626–2.205]				
**pN+**	***n* = 73**			0.14			
Yes vs No		1.613	[0.845–3.080]				
**ypT Stage**	***n* = 74**			**0.0045**	2.64	[1.160–6.031]	**0.02**
3–4 vs 0–2		3.27	[1.444–7.404]				
**ypN Stage**	***n* = 72**			0.29			
1–2 vs 0		1.419	[0.740–2.719]				
**TRG ^1^**	***n* = 73**			0.21			
3–5 vs 1–2		1.579	[0.771–3.235]				
**Median PD-L1 Biopsy**	***n* = 74**			0.10			
>0.15 vs ≤0.15		0.596	[0.317–1.119]				
**Median PD-L1 Surgery**	***n* = 74**			0.05			
>0.5 vs ≤0.5		0.542	[0.287–1.022]				
**Delta PD-L1 ^2^**	***n* = 74**			0.90			
>0.2 vs ≤0.2		0.962	[0.521–1.777]				
**Evolution of PD-L1+ Cells Rate**	***n* = 74**			0.07			
High-to-high vs low-to-low		0.329	[0.127–0.856]				
High-to-low vs low-to-low		1.154	[0.513–2.594]				
Low-to-high vs low-to-low		1.007	[0.450–2.251]				
High-to-high vs other		0.317	[0.132–0.758]	**0.0098**	0.33	[0.136–0.811]	**0.01**
**PD-L1 Biopsy**	***n* = 74**			**0.0065**			
>0.08 vs ≤0.08		0.422	[0.227–0.786]				

^1^ tumor regression grade; ^2^ Delta PD-L1 represents PD-L1+ cells rate after treatment minus PD-L1+ cells rate before treatment; ^3^ hazard ratio; ^4^ confidence interval; ^5^ Univariate and multivariate Cox proportional hazards regressions were used to estimate hazard ratio with its 95% confidence intervaI. Variables with a *p* < 0.20 in univariate analysis were included in the multivariate model. Tests were two-sided and a *p*-value less than 5% was considered statistically significant.

**Table 5:** Relation between clinical variables at baseline and after treatment and progression-free survival.

		Univariate Analysis			Multivariate Analysis		
Variable		HR ^3^	95% CI ^4^	*p* Value ^5^	HR	95% CI	*p* Value ^5^
**Gender**	***n* = 74**			0.83			
Female vs Male		0.937	[0.508–1.728]				
**Age (Years)**	***n* = 74**			0.85			
>69 vs ≤69		0.945	[0.522–1.710]				
**Tumor Distance from Anal Verge (cm)**	***n* = 72**			0.21			
≥10 cm vs <5 cm		1.628	[0.684–3.874]				
[5,6,7,8,9,10] cm vs <5 cm		1.878	[0.929–3.798]				
**Histological Subtype**	***n* = 70**			0.81			
Highly differentiated vs Poorly or Moderately differentiated adenocarcinoma		0.824	[0.442–1.538]				
Other vs Poorly or Moderately differentiated adenocarcinoma		1.056	[0.249–4.484]				
**cN Stage**	***n* = 61**			0.17			
N+ vs N0		1.571	[0.815–3.029]				
**Radiation Dose Per Fraction (Gy)**	***n* = 74**			0.30			
>2 vs ≤2		1.373	[0.752–2.507]				
**Chemotherapy**	***n* = 73**			0.71			
Yes vs No		1.119	[0.617–2.030]				
**Concurrent Chemotherapy**	***n* = 71**			0.89			
Yes vs No		1.048	[0.536–2.051]				
**Adjuvant Chemotherapy**	***n* = 72**			0.12			
Yes vs No		1.658	[0.869–3.163]				
**Time between End of Radiotherapy and Surgery (Days)**	***n* = 74**			0.34			
>40 vs ≤40		1.329	[0.739–2.392]				
**Conservative Surgery**	***n* = 73**			0.63			
Yes vs No		1.159	[0.633–2.123]				
**pN+**	***n* = 73**			0.13			
Yes vs No		1.611	[0.866–2.995]				
**ypT Stage**	***n* = 74**			**0.0092**			
3–4 vs 0–2		2.656	[1.274–5.537]		2.223	[1.055–4.684]	**0.03**
**ypN Stage**	***n* = 72**			0.36			
1–2 vs 0		1.336	[0.715–2.494]				
**TRG ^1^**	***n* = 73**			0.16			
3–5 vs 1–2		1.63	[0.823–3.229]				
**Median PD-L1 Biopsy**	***n* = 74**			0.03			
>0.15 vs ≤0.15		0.512	[0.279–0.938]				
**Median PD-L1 Surgery**	***n* = 74**			0.14			
>0.5 vs ≤0.5		0.645	[0.355–1.171]				
**Delta PD-L1 ^2^**	***n* = 74**			0.84			
>0.2 vs ≤0.2		1.062	[0.591–1.907]				
**Evolution of PD-L1+ Cells Rate**	***n* = 74**			0.06			
High-to-high vs low-to-low		0.344	[0.141–0.842]				
High-to-low vs low-to-low		0.914	[0.411–2.032]				
Low-to-high vs low-to-low		1.158	[0.546–2.456]				
High-to-high vs other		0.338	[0.150–0.760]	**0.0087**	0.402	[0.177–0.916]	**0.03**
**PD-L1 Biopsy**	***n* = 4**			**0.0037**			
>0.08 vs ≤0.08		0.414	[0.229–0.751]				

^1^ tumor regression grade; ^2^ Delta PD-L1 represents PD-L1+ cells rate after treatment minus PD-L1+ cells rate before treatment; ^3^ hazard ratio; ^4^ confidence interval; ^5^ Univariate and multivariate Cox proportional hazards regressions were used to estimate hazard ratio with its 95% confidence intervaI. Variables with a *p* < 0.20 in univariate analysis were included in the multivariate model. Tests were two-sided and a *p*-value less than 5% was considered statistically significant.

## Supplementary Materials

The following are available online at https://www.mdpi.com/2073-4409/9/9/2071/s1, Table S1: Tumor regression grade according to PD-L1 expression.
