# Supplementary material for: Radiotherapy Scheme Effect on PD-L1 Expression for Locally Advanced Rectal Cancer
Source: Cells. 2020 Sep 10;9(9):2071. doi: 10.3390/cells9092071 (PMC7563314; doi:10.3390/cells9092071)
Supplement: Supplementary file 1 [file cells-09-02071-s001.pdf]

Table S1. Tumor regression grade (TRG) according to PD-L1 expression

Tumor regression grade (TRG) according to median PD-L1+ cells rate on biopsy

	PD-L1 ≤ 0.15	PD-L1 > 0.15	p value
**TRG**			0.58
1	2 (5.6%)	2 (5.4%)	
2	9 (25.0%)	9 (24.3%)	
3	9 (25.0%)	15 (40.5%)	
4	13 (36.1%)	10 (27.0%)	
5	3 (8.3%)	1 (2.7%)	
Missing	1	0	

Tumor regression grade (TRG) according to median PD-L1+ cells rate on surgical specimen

	PD-L1≤ 0.5	PD-L1 > 0.5	p value
**TRG**			0.21
1	3 (8.1%)	1 (2.8%)	
2	11 (29.7%)	7 (19.4%)	
3	11 (29.7%)	13 (36.1%)	
4	12 (32.4%)	11 (30.6%)	
5	0 (0.0%)	4 (11.1%)	
Missing	0	1	

Tumor regression grade (TRG) according to deltaPD-L1+ cells rate

	Delta ≤ 0.2	Delta > 0.2	p value
**TRG**			0.38
1	2 (5.4%)	2 (5.6%)	
2	10 (27.0%)	8 (22.2%)	
3	13 (35.1%)	11 (30.6%)	
4	12 (32.4%)	11 (30.6%)	
5	0 (0.0%)	4 (11.1%)	
Missing	0	1	

Tumor regression grade (TRG) according to median PD-L1+ cells rate evolution profile

	High-to-high	High-to-low	Low-to-high	Low-to-low	p value
**TRG**					0.51
1	1 (4.8%)	1 (6.3%)	0 (0.0%)	2 (9.5%)	
2	5 (23.8%)	4 (25.0%)	2 (13.3%)	7 (33.3%)	
3	8 (38.1%)	7 (43.8%)	5 (33.3%)	4 (19.0%)	
4	6 (28.6%)	4 (25.0%)	5 (33.3%)	8 (38.1%)	
5	1 (4.8%)	0 (0.0%)	3 (20.0%)	0 (0.0%)	

Missing	0	0	1	0	
